# Serum GFAP, NfL, and tau concentrations are associated with worse neurobehavioral functioning following mild, moderate, and severe TBI: a cross-sectional multiple-cohort study

**DOI:** 10.3389/fneur.2023.1223960

**Published:** 2024-01-16

**Authors:** Katie A. Edwards, Rael T. Lange, Sara M. Lippa, Tracey A. Brickell, Jessica M. Gill, Louis M. French

**Affiliations:** ^1^School of Nursing, Johns Hopkins University, Baltimore, MD, United States; ^2^Traumatic Brain Injury Center of Excellence, Silver Spring, MD, United States; ^3^Walter Reed National Military Medical Center, Bethesda, MD, United States; ^4^National Intrepid Center of Excellence, Bethesda, MD, United States; ^5^General Dynamics Information Technology, Silver Spring, MD, United States; ^6^Department of Psychiatry, University of British Columbia, Vancouver, BC, Canada; ^7^Department of Physical Medicine and Rehabilitation, Uniformed Services University of the Health Sciences, Bethesda, MD, United States; ^8^Department of Neuroscience, Uniformed Services University of the Health Sciences, Bethesda, MD, United States

**Keywords:** traumatic brain injury, tau, glial fibrillary acidic protein (GFAP), neurofilament light (NfL), military, neurobehavior

## Abstract

**Introduction:**

The purpose of this study was to examine whether blood-based biomarkers associate with neurobehavioral functioning at three time points following traumatic brain injury (TBI).

**Materials and methods:**

Participants were 328 United States service members and veterans (SMVs) prospectively enrolled in the Defense and Veterans Brain Injury Center-Traumatic Brain Injury Center of Excellence (DVBIC-TBICoE) 15-Year Longitudinal TBI Study, recruited into three groups: uncomplicated mild TBI (MTBI, *n* = 155); complicated mild, moderate, severe TBI combined (STBI, *n* = 97); non-injured controls (NIC, *n* = 76). Participants were further divided into three cohorts based on time since injury (≤12 months, 3–5 years, and 8–10 years). Participants completed the Minnesota Multiphasic Personality Inventory-2-Restructured Format (MMPI-2-RF) and underwent blood draw to measure serum concentrations of glial fibrillary acidic protein (GFAP), neurofilament light (NfL), and tau. A total of 11 MMPI-2-RF scales were examined (e.g., depression, anxiety, anger, somatic, cognitive symptoms). Stepwise hierarchical regression models were conducted within each group.

**Results:**

Significant associations were found between biomarkers and MMPI-2-RF scales (all *p* < 0.05; *R^2^*Δ > 0.10). GFAP was inversely related to (a) neurological complaints in the MTBI group at ≤12 months, (b) demoralization, anger proneness in the STBI group at ≤12 months, and (c) head pain complaints in the STBI group at 8–10 years. NfL was (a) related to low positive emotions in the NIC group; and inversely related to (b) demoralization, somatic complaints, neurological complaints, cognitive complaints in the MTBI group at ≤12 months, (c) demoralization in the STBI group at ≤12 months, and (d) demoralization, head pain complaints, stress/worry in the STBI group at 3–5 years. In the STBI group, there were meaningful findings (*R^2^*Δ > 0.10) for tau, NFL, and GFAP that did not reach statistical significance.

**Discussion:**

Results indicate worse scores on some MMPI-2-RF scales (e.g., depression, stress/worry, neurological and head pain complaints) were associated with lower concentrations of serum GFAP, NfL, and tau in the sub-acute and chronic phase of the recovery trajectory up to 5 years post-injury, with a reverse trend observed at 8–10 years. Longitudinal studies are needed to help elucidate any patterns of association between blood-based biomarkers and neurobehavioral outcome over the recovery trajectory following TBI.

## 1 Introduction

Traumatic brain injury (TBI) is common among U.S. Service Members and Veterans (SMVs), with 450,000 SMVs experiencing a TBI over the past two decades (1). Although many patients with mild, moderate, and severe TBIs experience good recoveries, some patients report long-term neurobehavioral symptoms in the months and years following injury (2), including SMVs (3–5). Blood-based biomarkers are a promising avenue to identifying patients that may be at risk of poor long-term neurobehavioral outcomes. Identifying blood-based biomarkers that relate to long-term neurobehavioral functioning may also support understanding of neuropathological processes and development of interventions.

Neuronal and glial blood biomarkers have been examined previously and shown to be correlated to TBI severity and symptoms (6, 7). Neurofilament light (NfL), a protein highly expressed in long myelinated axons, is indicative of axonal injury and is linked to post-concussive symptoms after TBI in athletes engaged in contact sports (8, 9). NfL is also associated with chronic neurobehavioral symptoms in military populations (10, 11), and is associated with worse cognitive function in military personnel with mild TBI (12). Additionally, elevations of CSF and blood NfL levels have been observed in several neuropsychiatric conditions, especially when determined by an organic brain injury (13). Another marker of axonal injury, tau, is a microtubule-associated protein that forms neurofibrillary tangles when hyperphosphorylated. Tau is a protein linked to cognitive impairment and neurodegeneration, and is implicated in Alzheimer’s disease (14, 15). Additionally, tau is associated with posttraumatic stress disorder and neurocognitive decline following TBI (12, 16, 17).

Glial fibrillary acidic protein (GFAP), comprises the cytoskeleton of astrocytes within the central nervous system, and is implicated in neurodegenerative diseases (18, 19). Following TBI, reactive astrocytes have an important role in initiating pro-and anti-inflammatory immune mediators and regulating blood–brain barrier response. As a marker for reactive astrocytes, GFAP is observed to be elevated after TBI and is associated with abnormal neuroimaging (7) as well as cognitive decline in military personnel with severe TBI (20).

Examination of these biomarkers in the chronic phase following TBI, and their potential relationship to neurobehavioral outcomes is essential in understanding their impact in recovery trajectories. A longitudinal study in the same population as this study showed an association with elevated baseline biomarkers (<12 months, NfL, tau, GFAP, UCHL-1) and chronic neurobehavioral symptoms (21), but did not examine biomarker levels over chronic, cross-sectional time points. While recent work has examined chronic trajectories of neuronal and glial blood biomarkers up to 5 years following injury (10, 22), the majority of research has focused on measuring biomarkers at acute and subacute time points, and less is known regarding the relation of these biomarkers to neurobehavioral outcomes in chronic TBI. The purpose of this cross-sectional multi-cohort study was to examine whether blood-based biomarkers NfL, GFAP, and tau associate with neurobehavioral functioning at three time points following TBI.

## 2 Materials and methods

### 2.1 Participants

Participants were 328 United States service members and veterans (SMVs) prospectively enrolled in the Defense and Veterans Brain Injury Center-Traumatic Brain Injury Center of Excellence (DVBIC-TBICoE) 15-Year Longitudinal TBI Study. The study was approved by the Institutional Review Board of Walter Reed National Medical Military Center, and all participants provided written informed consent. General inclusion criteria for the larger study included active-duty service members or veterans eligible to receive military benefits, with lifetime history of TBI or no lifetime history of TBI (non-injured control group, NIC). Participants were included in the NIC group if they had no history of an orthopedic and/or soft-tissue injury, and no history of TBI. Participants were included in the TBI group if they had evidence of a TBI that was the result of a combat or non-combat related event as indicated by one or more of the following: (i) established period of alteration of consciousness (AOC), loss of consciousness (LOC), or post-traumatic amnesia (PTA) that was directly attributable to head trauma; (ii) trauma-related intracranial abnormalities as indicated by CT or MRI scan; and/or (iii) Glasgow Coma Scale (GCS) < 15 (if available). General exclusion criteria included: non-English speaking; history of significant neurological conditions; history of pervasive formal psychiatric diagnosis prior to the military; diagnosis of psychotic disorder, personality disorder, or bipolar disorder; and learning disability or attention deficit hyperactivity disorder only in cases of persistent impact on academic performance.

For the purposes of this study, participants were selected from a larger sample of 999 participants who had completed a blood draw. Participants were included in the final analyses if they had at least one useable biomarker sample for the NIC group or, for the TBI groups, MRI results and at least one useable biomarker sample within the specified periods following TBI (≤12 months, 3–5 years, and 8–10 years), had completed the Minnesota Multiphasic Personality Inventory-2-Restructured Format (MMPI-2-RF), and passed symptom validity tests. Blood draws and the MMPI-2-RF were collected on the same day for each participant. The final sample (*N* = 328) was separated into three groups: uncomplicated mild TBI (MTBI, *n* = 155), complicated mild, moderate, and severe TBI combined (STBI, *n* = 97), and non-injured controls (NIC, *n* = 76). Participants in the TBI groups were further subdivided into three cohorts based on time since injury [≤12 months (NIC, *n* = 76; MTBI, *n* = 46; STBI, *n* = 41), 3–5 years (NIC, *n* = 76; MTBI, *n* = 57; STBI, *n* = 30), and 8–10 years (NIC, *n* = 76; MTBI, *n* = 52; STBI, *n* = 26)]. The same control group (NIC) was used as a comparison for each TBI group.

### 2.2 TBI evaluation and classification

TBI diagnosis and classification have been described in detail previously (23). Briefly, classification of TBI severity by consensus was determined through comprehensive interview (24) and case-conferencing of all potential lifetime TBI events combined with medical records. TBI severity was classified as follows: (a) uncomplicated mild TBI: (i) GCS = 13–15, PTA < 24 h, LOC < 30 min, and/or AOC present, and (ii) no trauma-related intracranial abnormality on CT or MRI; (b) complicated mild TBI: (i) GCS = 13–15, PTA < 24 h, LOC < 30 min, and/or AOC present, and (ii) trauma-related intracranial abnormality on CT or MRI; (c) moderate TBI: LOC > 30 min-24 h, PTA 1–7 days, and ICA present or absent; (d) severe TBI: LOC > 24 h, PTA >7 days, and ICA present or absent.

### 2.3 Laboratory analyses

Participants underwent non-fasting blood draw to measure serum concentrations of GFAP, NfL, and tau. Blood samples were collected with plastic lithium heparin tubes, processed within 1 h of collection, and stored at −80°C until batch assay analysis using Simoa™ (Quanterix, Lexington, MA, United States), a high-definition-1 analyzer, single-molecule array technology. The samples for GFAP, NfL, and tau assays were randomized and run in duplicate with laboratory personnel blinded to participant groups. The coefficient of variation (CV) for all concentration values were < 20%. Average CVs were 3.5% for GFAP, 6.3% for NfL, and 14.4% for tau. The lower limit of quantification (LLOQ) for the assays are 0.467 pg/mL for GFAP, 0.241 pg/mL for NFL, and 0.053 pg/mL for tau.

### 2.4 Psychological assessment

Participants completed the MMPI-2-RF, a measure designed to evaluate psychological symptomatology (25). For this study, only 11 MMPI-2-RF scales were selected for examination based on symptoms that are commonly reported following TBI: Demoralization (RCd), Somatic Complaints (RC1), Low Positive Emotions (RC2), Malaise (MLS), Head Pain Complaints (HPC), Neurological Complaints (NUC), Cognitive Complaints (COG), Stress/Worry (STW), Anxiety (ANX), Anger Proneness (ANP), and Aggression (AGG). In addition, the validity scales were used for the purposes of evaluating symptom validity. Based on recommended cutoff scores, participants were not included if they were considered to have exaggerated symptoms on the MMPI-2-RF (i.e., F-r ≥ 100 T or Fp-r ≥ 90 T or Fs ≥ 100 T or FBS-r ≥ 100 T or RBS ≥ 100 T) or whose scores were not considered interpretable (i.e., Cannot Say scores > 14, or VRIN-r/TRIN-r scores > 79 T).

### 2.5 Data analyses

Demographic and injury characteristics were compared between groups (NIC, MTBI, and STBI) at each time point using analysis of variance (ANOVA; i.e., demographics, MMPI-2-RF scales) or Kruskal-Wallis H tests (i.e., biomarkers) for continuous variables and Chi-square analyses for categorical variables (i.e., demographic measures). Pairwise comparisons were undertaken using ANOVA followed by *post hoc* analysis (i.e., demographics, MMPI-2-RF scales) or Mann–Whitney *U* tests (i.e., biomarkers) for continuous variables and Chi-square analyses for categorical variables. Spearman’s rho correlation analysis (continuous variables) and Mann–Whitney *U* tests (categorical variables) were used to evaluate the relationship between the three biomarkers and demographic and injury characteristics in the entire group. Demographic or injury variables that were meaningfully associated with a biomarker (r_s_ ≥ 0.25 for continuous variables, or *p* < 0.05 for categorical variables) were used as covariates in subsequent analyses. A series of hierarchical regression analyses were undertaken (controlling for relevant covariates), to examine whether each of the three biomarkers could predict MMPI-2-RF scores. Due to small sample sizes, a clinically meaningful relationship between a biomarker and symptom change score in the regression analyses was defined as either (a) *p* < 0.05 or (b) R^2^/R^2^∆ > 0.10 (i.e., percent variance accounted for >10%). The criterion for R^2^/R^2^∆ was chosen because statistically significant results using regression analyses are routinely found in larger samples when R^2^/R^2^∆ < 0.10 (20, 26). Tests were 2-sided with a significance threshold of *p* < 0.05. Statistical analyses were conducted with SPSS Version 24.0 (Armonk, NY: IBM Corp.), and figures were created using GraphPad Prism version 9.3.1 (La Jolla, CA: GraphPad Software).

## 3 Results

Descriptive statistics and group comparisons for demographic and injury characteristics, and MMPI-2-RF scores, in each of the three cohorts is presented in Tables 1–3. In the ≤12 months post-TBI cohort (Table 1), the MTBI and STBI groups were significantly younger than the NIC group (*p* < 0.001). There was a significant difference in the proportion of men in the STBI group (97.6%) compared to the NIC group (77.6%; *p* = 0.014). In the 3–5 years post-TBI cohort (Table 2), the STBI group was significantly younger than the NIC group (*p* < 0.001). There was a significant difference in the proportion of men in the MTBI group (94.7%) compared to the NIC group (77.6%; *p* = 0.015). In the 8–10 years post-TBI cohort (Table 3), there were no significant differences in age between groups. However, there was a significantly higher proportion of men in the MTBI group (96.2%) and the STBI group (100%) compared to the NIC group (77.6%; *p* < 0.001). In addition, for race, there was a higher proportion of participants in the MTBI (91.7%) and STBI groups (96.0%) classified as white compared to the NIC group (71.6%; *p* = 0.018). The median number of deployments was higher in the MTBI group (3.0) as compared to the STBI (2.0) and NIC groups (1.0; *p* < 0.001). In all cohorts, the MTBI and STBI groups had fewer years of education than the NIC group (*p* < 0.001).

**Table 1:** Demographic and injury characteristics, ≤12 months post-TBI.

	NIC (*n* = 76)		MTBI (*n* = 46)		STBI (*n* = 41)				
	M	SD	M	SD	M	SD	*F*	*p*	Pairwise comparisons < 0.05
Age in years	39.8	10.8	29.5	8.0	30.2	9.1	21.52	<0.001	1 > 2,3
Years of education	16.3	2.4	14.3	2.1	14.0	2.0	18.06	<0.001	1 > 2,3
RCd	49.8	10.0	51.4	11.2	48.1	8.9	1.11	0.333	-
RC1	55.2	12.6	58.7	8.8	58.5	10.6	1.87	0.157	-
RC2	48.6	11.2	51.2	11.0	48.9	9.2	0.93	0.398	-
MLS	51.9	11.1	58.8	11.8	59.3	10.9	8.12	<0.001	1 < 2,3
HPC	53.8	12.3	55.6	11.6	56.1	11.6	0.613	0.543	-
NUC	55.5	12.5	62.0	10.4	61.7	12.1	5.97	0.003	1 < 2,3
COG	56.1	13.9	61.4	14.4	57.0	15.0	2.08	0.129	-
STW	51.3	11.7	50.0	10.2	47.5	10.0	1.61	0.203	-
AXY	52.5	13.9	50.9	11.4	53.1	13.8	0.334	0.717	-
ANP	49.9	9.8	52.0	10.8	52.0	11.6	0.803	0.450	-
AGG	46.6	9.0	50.2	12.0	50.5	10.9	2.72	0.069	-
	**Med**	**IQR**	**Med**	**IQR**	**Med**	**IQR**	**K-W**	***p***	**M-W, *p* < 0.05**
Number of TBIs	NA	NA	1.0	0.00	1.0	0.00		0.148	-
Number of deployments	1.0	3.0	2.0	2.25	2.0	2.0	1.020	0.601	-
Tau	0.49	1.16	0.43	1.52	0.43	2.40	3.934	0.140	-
NfL	8.05	4.27	6.55	8.72	16.83	24.21	22.068	<0.001	1,2 < 3
GFAP	89.55	55.68	61.39	43.79	66.65	38.58	30.624	<0.001	1 > 2,3
	***n***	**%**	***n***	**%**	***n***	**%**	**Chi square**	***p***	**Pairwise comparisons < 0.05**
White	48	71.6	34	82.9	34	89.4	7.347	0.692	-
Non-Hispanic	66	86.8	40	87.0	38	92.7	6.726	0.347	-
Men	59	77.6	40	87.0	40	97.6	8.569	0.014	1 < 3

For pairwise comparisons and M-W, groups are 1 = NIC; 2 = MTBI, and 3 = STBI. AGG, Aggression; ANP, Anger Proneness; AXY, Anxiety; COG, Cognitive Complaints; GFAP, glial fibrillary acidic protein; HPC, Head Pain Complaints; K-W, Kruskal-Wallis; M-W, Mann–Whitney; MTBI, uncomplicated mild TBI; MLS, Malaise; NfL, neurofilament light; NIC, non-injured controls; NUC, Neurological Complaints; RC1, Somatic Complaints; RC2, Low Positive Emotions; RCd, Demoralization; STBI, complicated mild, moderate, and severe TBI; STW, Stress/Worry.

**Table 2:** Demographic and injury characteristics, 3–5 years post-TBI.

	NIC (*n* = 76)		MTBI (*n* = 57)		STBI (*n* = 30)				
	M	SD	M	SD	M	SD	*F*	*p*	Pairwise comparisons < 0.05
Age in years	39.8	10.8	35.9	8.9	34.5	9.9	4.17	0.017	1 > 3
Years of education	16.3	2.4	15.2	2.1	14.7	2.0	6.44	0.002	1 > 2,3
RCd	49.8	10.0	52.9	10.5	51.7	10.4	1.57	0.211	-
RC1	55.2	12.6	62.1	13.8	57.8	10.6	4.76	0.010	1 < 2
RC2	48.6	11.2	50.9	11.3	51.2	12.1	0.96	0.384	-
MLS	51.9	11.1	59.2	13.3	57.9	14.5	6.102	0.003	1 < 2
HPC	53.8	12.3	59.7	13.8	57.8	13.4	3.501	0.032	1 < 2
NUC	55.5	12.5	63.9	13.6	56.8	13.1	7.28	0.001	1 < 2
COG	56.1	13.9	65.2	16.0	61.9	10.6	6.92	0.001	1 < 2
STW	51.3	11.7	51.9	12.7	49.5	8.5	0.44	0.643	-
AXY	52.5	13.9	59.4	17.7	54.4	11.9	3.54	0.031	1 < 2
ANP	49.9	9.8	54.2	11.2	54.2	12.2	3.33	0.038	-
AGG	46.6	9.0	52.6	11.4	50.8	13.5	5.4	0.005	1 < 2
	**Med**	**IQR**	**Med**	**IQR**	**Med**	**IQR**	**K-W**	***p***	**M-W, *p* < 0.05**
Number of TBIs	NA	NA	1.0	0.00	1.0	0.00		0.269	-
Number of deployments	1.0	3.0	2.0	2.0	2.0	2.0	4.864	0.088	-
Tau	0.49	1.16	0.42	0.29	0.40	0.31	5.962	0.051	-
NfL	8.05	4.27	6.30	3.90	6.46	5.37	14.555	0.001	1 > 2,3
GFAP	89.55	55.68	66.31	34.23	84.20	68.59	13.847	0.001	1 > 2
	***n***	**%**	***n***	**%**	***n***	**%**	**Chi square**	***p***	**Pairwise comparisons < 0.05**
White	48	71.6	45	84.9	22	84.6	12.467	0.409	-
Non-Hispanic	66	86.8	51	89.5	25	83.3	10.662	0.099	-
Men	59	77.6	54	94.7	27	90.0	8.376	0.015	1 < 2

For pairwise comparisons and M-W, groups are 1 = NIC; 2 = MTBI, and 3 = STBI. AGG, Aggression; ANP, Anger Proneness; AXY, Anxiety; COG, Cognitive Complaints; GFAP, glial fibrillary acidic protein; HPC, Head Pain Complaints; K-W, Kruskal-Wallis; M-W, Mann–Whitney; MTBI, uncomplicated mild TBI; MLS, Malaise; NfL, neurofilament light; NIC, non-injured controls; NUC, Neurological Complaints; RC1, Somatic Complaints; RC2, Low Positive Emotions; RCd, Demoralization; STBI, complicated mild, moderate, and severe TBI; STW, Stress/Worry.

**Table 3:** Demographic and injury characteristics, 8–10 years post-TBI.

	NIC (*n* = 76)		MTBI (*n* = 52)		STBI (*n* = 26)				
	M	SD	M	SD	M	SD	*F*	*p*	Pairwise comparisons < 0.05
Age in years	39.8	10.8	40.3	8.2	38.0	7.6	0.53	0.591	-
Years of education	16.3	2.4	14.9	2.1	14.7	2.6	7.04	0.001	1 > 2,3
RCd	49.8	10.0	54.8	9.0	55.3	12.3	5.06	0.007	1 < 2
RC1	55.2	12.6	63.5	12.2	63.9	12.3	8.84	<0.001	1 < 2,3
RC2	48.6	11.2	53.7	11.5	55.6	12.1	5.13	0.007	1 < 2,3
MLS	51.9	11.1	60.2	11.6	62.4	13.0	12.04	<0.001	1 < 2,3
HPC	53.8	12.3	61.5	12.7	59.6	10.6	6.72	0.002	1 < 2
NUC	55.5	12.5	65.4	14.2	68.4	15.7	12.72	<0.001	1 < 2,3
COG	56.1	13.9	66.3	13.3	68.7	13.0	12.97	<0.001	1 < 2,3
STW	51.3	11.7	52.8	10.0	50.5	9.1	0.46	0.633	-
AXY	52.5	13.9	57.6	14.6	53.0	13.0	2.17	0.118	-
ANP	49.9	9.8	52.8	10.7	54.4	10.1	2.47	0.088	-
AGG	46.6	9.0	52.7	11.4	48.6	10.3	5.75	0.004	1 < 2
	**Med**	**IQR**	**Med**	**IQR**	**Med**	**IQR**	**K-W**	***p***	**M-W, *p* < 0.05**
Number of TBIs	NA	NA	1.0	1.0	1.0	2.0		0.608	-
Number of deployments	1.0	3.0	3.0	3.0	2.0	2.0	16.705	<0.001	1 < 2 > 3
Tau	0.49	1.16	0.42	0.35	0.40	0.27	5.345	0.069	-
NfL	8.05	4.27	8.00	5.12	7.83	4.87	0.521	0.771	-
GFAP	89.55	55.68	71.46	39.47	96.78	63.06	9.903	0.007	1 > 2 < 3
	***n***	**%**	***n***	**%**	***n***	**%**	**Chi square**	***p***	**Pairwise comparisons < 0.05**
White	48	71.6	44	91.7	24	96.0	24.442	0.018	1 < 2,3
Non-Hispanic	66	86.8	45	86.5	25	96.2	3.038	0.804	-
Men	59	77.6	50	96.2	26	100	14.196	0.001	1 < 2,3

For pairwise comparisons and M-W, groups are 1 = NIC; 2 = MTBI, and 3 = STBI. AGG, Aggression; ANP, Anger Proneness; AXY, Anxiety; COG, Cognitive Complaints; GFAP, glial fibrillary acidic protein; HPC, Head Pain Complaints; K-W, Kruskal-Wallis; M-W, Mann–Whitney; MTBI, uncomplicated mild TBI; MLS, Malaise; NfL, neurofilament light; NIC, non-injured controls; NUC, Neurological Complaints; RC1, Somatic Complaints; RC2, Low Positive Emotions; RCd, Demoralization; STBI, complicated mild, moderate, and severe TBI; STW, Stress/Worry.

At ≤12 months post-TBI, there were significant main effects for MLS and NUC. Pairwise comparisons revealed that the MTBI and STBI groups had significantly higher scores compared to the NIC group. At 3–5 years post-TBI, there were significant main effects for RC1, MLS, HPC, NUC, COG, AXY, and AGG. Pairwise comparisons revealed that the MTBI group reporting significantly higher scores on all measures compared to the NIC group. At 8–10 years post-TBI, there were significant main effects for RCd, HPC, and AGG, with the MTBI group reporting significantly higher scores compared to the NIC group; and for RC1, RC2, MLS, NUC, and COG, so that the MTBI and STBI groups had significantly higher scores as compared to the NIC group.

**Figure 1:**
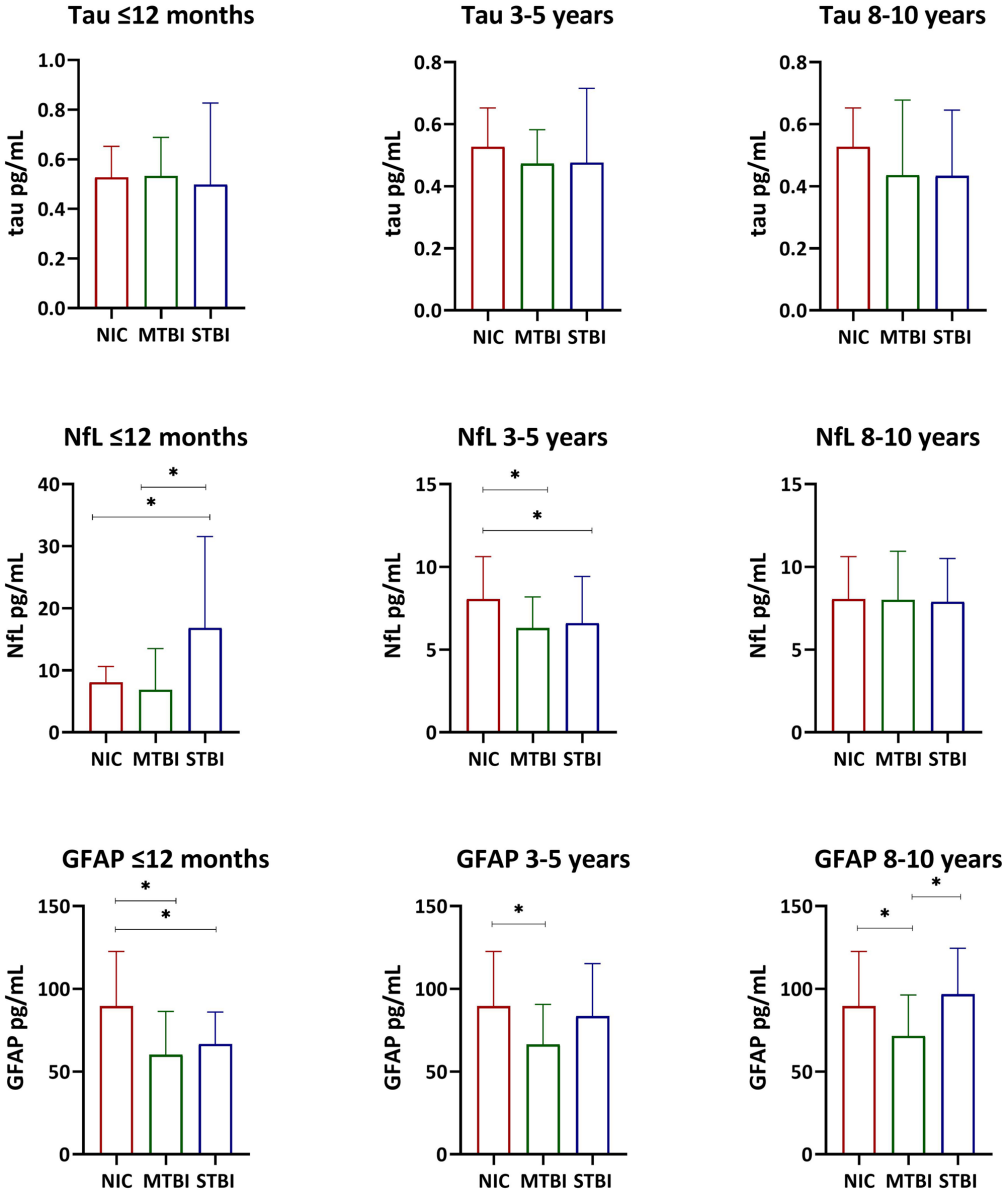
Serum Tau, NFL, and GFAP Concentrations. Bar graphs display median and interquartile range. The Kruskal-Wallis test and the Mann-Whitney U test were used to examine differences in tau, NfL, and GFAP serum concentrations within each group. *p < 0.05.

Descriptive statistics and group comparisons for GFAP, NfL, and tau concentrations in each of the three cohorts is also presented in Tables 1–3. Differences in biomarker concentrations within each group is further presented in Figure 1. In the ≤12 months post-TBI cohort, NfL was higher in the STBI group compared to the MTBI and NIC groups (*p* < 0.001); GFAP was lower in the MTBI and STBI groups compared to the NIC group (*p* < 0.001). In the 3–5 years post-TBI cohort, NfL was lower in the MTBI and STBI groups compared to the NIC group (*p* < 0.001); GFAP was lower in the MTBI group compared to the NIC group (*p* = 0.001). In the 8–10 years post-TBI cohort, GFAP was significantly lower in the MTBI group compared to the STBI and NIC groups (*p* = 0.007). There were no significant differences in tau concentrations between groups in any cohort.

Spearman’s rho correlations and Mann Whitney *U* tests examining the association between select demographic and injury variables with baseline biomarkers in the entire sample are presented in Table 4. There were significant correlations (*p* < 0.05) between GFAP and education (r_s_ = 0.28), age (r_s_ = 0.24), number of TBIs (r_s_ = −0.17), time since injury (r_s_ = 0.22), and TBI severity (r_s_ = −0.11); NFL with deployments (r_s_ = 0.13), education (r_s_ = 0.13), and age (r_s_ = 0.28); and tau with deployments (r_s_ = −0.12) and number of TBIs (r_s_ = −0.12). Gender was significantly associated with tau (*p* = 0.031) and GFAP (*p* = 0.036). Note that only those variables that were meaningfully associated with the biomarkers (r_s_ > 0.25 for continuous variables, or p < 0.05 for categorical variables) were selected as covariates in subsequent analyses.

**Table 4:** Spearman rho correlation coefficients and Mann–Whitney *U*-tests comparing demographic and injury variables with baseline biomarkers.

	Tau	NfL	GFAP
	r_s_	r_s_	r_s_
Age	0.02	0.28^**^	0.24^**^
Education	0.09	0.13^**^	0.28^**^
Number of deployments	−0.12^*^	0.13^*^	−0.03
Time since injury	0.02	−0.04	0.22^**^
Number of TBI	−0.12^*^	−0.05	−0.17^**^
TBI severity	−0.09	−0.07	−0.11^*^
	***p***	***p***	***p***
Gender (male/female)	0.031	0.875	0.036
Race (white/non-white)	0.570	0.145	0.141
Ethnicity (Hispanic/non-Hispanic)	0.839	0.738	0.378

*N* = 328 (NIC = 76, MTBI = 155, STBI = 97). GFAP, Glial Fibrillary Acidic Protein; MTBI, uncomplicated mild TBI; NfL, Neurofilament Light; NIC, non-injured control; STBI, uncomplicated mild, moderate, and severe TBI combined; TBI, traumatic brain injury. ^*^p < 0.05, ^**^p < 0.001.

A summary of the linear regression analysis results (controlling for relevant covariates) using the biomarkers to predict MMPI-2-RF scores within the TBI severity groups is presented in Table 5. Serum GFAP was inversely related to NUC in the MTBI group at ≤12 months post-injury. In the STBI group, GFAP was also inversely related to RCd and ANP at ≤12 months post-injury, and inversely related to HPC at 8–10 years post-injury. Serum NfL was significantly related to RC2 in the NIC group. In the MTBI group at ≤12 months post-injury, NfL was inversely related to RCd, RC1, NUC, and COG. In the STBI group at ≤12 months post-injury, NfL was inversely related to RCd. In the STBI group at 3–5 years post injury, NfL was inversely related to RCd, HPC, and STW. In addition, several findings in the STBI group had meaningful relationships (i.e., percent variance accounted for >10%). In the STBI group at 3–5 years post injury, NfL was inversely related to COG and tau was inversely related to STW. In the STBI group at 8–10 years post injury, NfL was related to AGG, GFAP was related to AGG, and tau was related to RCd and AXY.

**Table 5:** Summary of linear regression analysis results for biomarkers to predict MMPI-2-RF scores.

Group	Time point	Biomarker	MMPI-2-RF Scale	R^2^Δ	Beta	Sig.
NIC	n/a	NfL	RC2	0.074	0.273	0.017
MTBI	≤12 months	NfL	RCd	0.097	−0.311	0.035
			RC1	0.150	−0.388	0.008
			NUC	0.164	−0.405	0.005
			COG	0.117	−0.341	0.020
		GFAP	NUC	0.101	−0.318	0.031
STBI	≤12 months	NfL	RCd	0.106	−0.326	0.038
		GFAP	RCd	0.163	−0.403	0.009
			ANP	0.110	−0.332	0.034
STBI	3–5 years	NfL	RCd	0.235	−0.485	0.007
			HPC	0.168	−0.409	0.025
			COG^*^	0.101	−0.319	0.086
			STW	0.295	−0.543	0.002
		Tau	STW^*^	0.113	−0.336	0.070
STBI	8–10 years	NfL	AGG^*^	0.105	0.324	0.106
		GFAP	HPC	0.243	−0.493	0.011
			AGG^*^	0.106	0.325	0.105
		Tau	RCd^*^	0.144	0.380	0.055
			AXY^*^	0.109	0.330	0.100

AGG, Aggression; ANP, Anger Proneness; AXY, Anxiety; COG, Cognitive Complaints; GFAP, glial fibrillary acidic protein; HPC, Head Pain Complaints; MTBI, uncomplicated mild TBI; MLS, Malaise; NfL, neurofilament light; NIC, non-injured controls; NUC, Neurological Complaints; RC1, Somatic Complaints; RC2, Low Positive Emotions; RCd, Demoralization; STBI, complicated mild, moderate, and severe TBI; STW, Stress/Worry. ^*^R^2^Δ ≥ 10% in linear regression (not significant).

## 4 Discussion

This cross-sectional multi-cohort study examined GFAP, NfL, and tau in relation to neurobehavioral outcomes assessed with a well-validated personality inventory (MMPI-2-RF) with and without a TBI at ≤12 months, 3–5 years, and 8–10 years post-injury. After controlling for covariates, unexpectedly, worse MMPI-2-RF scores were associated with lower concentrations of serum GFAP, NfL, and tau, mostly at the ≤12 months and 3–5 year time points. Lower GFAP was significantly related to neurological complaints in participants with uncomplicated mild TBI ≤12 after injury. Lower GFAP also was related to demoralization and anger proneness in participants with complicated mild, moderate, and severe TBI at ≤12 months, and head pain complaints at 8–10 years following injury. Lower NfL was related to demoralization, somatic complaints, neurological complaints, and cognitive complaints in uncomplicated mild TBI at ≤12 months post-TBI. In addition, lower NfL was related to demoralization in participants with complicated mild, moderate, and severe TBI at ≤12 months and 3–5 years after injury, as well as head pain complaints, stress/worry, and cognitive complaints 3–5 years after injury. Tau was inversely related to stress/worry in STBI at 3–5 years post injury. When including clinically meaningful associations (i.e., *R^2^*Δ > 0.10), this trend reversed at 8–10 years, and increased tau was associated with worse demoralization and anxiety scores following STBI. Similarly, increased NfL and GFAP were associated with worse aggression scores in the STBI group at 8–10 years. These results suggest GFAP, NfL, and tau may be useful in understanding neurobehavioral outcomes beyond the acute recovery period, for up to 10 years following TBI.

GFAP, NfL, and tau have been implicated in progressing symptoms, neurodegeneration, and axonal injury (6, 10, 15). GFAP measured in plasma, combined with tau and NfL, are promising biomarkers in distinguishing patients with acute mTBI from controls, with higher biomarker concentrations indicating mTBI (27). Additionally, positive serum GFAP, together with UCHL-1, have been shown to have high sensitivity in predicting acute intracranial injuries detected by head computed tomography scans (28). Within a population of military veterans with a history of TBI, increased GFAP and NfL concentrations have been shown to differentiate participants with cognitive impairment when measured decades after TBI (29). In another population of SMVs with chronic TBI (median 6–9 years post-TBI), elevations in NfL correlate with increased severity of post-traumatic stress, post-concussive symptoms, and depressive symptoms (10).

However, findings in the current study are contradictory to the hypothesis that higher GFAP and NfL would be associated with worse neurobehavioral outcomes throughout the recovery trajectory. In a recent longitudinal biomarker study using the same population as the current study, elevated baseline biomarkers (NfL, GFAP, tau) within the first 12 months of injury were demonstrated to predict deteriorating MMPI-2-RF scores at 2+ years post-injury (21). Similarly in this population, elevated GFAP within 12 months of TBI predicted poor cognitive outcome (perceptual reasoning) following complicated mild and severe TBI (20), and elevated NfL and tau predicted cognitive decline (perceptual reasoning, executive functioning) following uncomplicated mild TBI (12). Unexpectedly, the current study demonstrates that decreased biomarker concentrations measured at chronic, cross-sectional time points in complicated mild, moderate, and severe TBI tend to be associated with worse MMPI-2-RF scores at 3–5 years post-TBI, with the reverse trend observed at the 8–10 year time point (i.e., increased biomarker concentrations associate with worse scores; with the exception of GFAP and HPC). Although this finding is contrary to the expected findings by the authors, biomarker concentrations that are initially elevated in the first 12 months post-injury are not necessarily expected to remain elevated at every chronic time point. One possible explanation is that decreasing chronic biomarker concentrations may indicate underlying impaired neurological pathways as the chronic stage of recovery progresses. To evaluate this explanation, further studies including longitudinal and within-subjects analyses are needed. In one study of the same population as the current study, NfL measured in extracellular vesicles (EVs) has shown similar inverse correlations with neuropsychological outcomes, although these correlations did not reach significance (30). A similar relationship between serum GFAP concentrations and emotional well-being was reported in female participants in a another population of SMVs with chronic TBI (median 6.4 years post-TBI), with higher GFAP associated with better emotional well-being in the female group (31). Although sex was controlled for in the current study, there was not enough statistical power to differentiate male and female groups, and further exploration of potential sex differences is warranted in future work.

In further support of recent literature, a study of subacute and chronic TBI in civilians showed a linear decrease in serum NfL over the first 5-years following mild to moderate TBI (22). However, in contrast to the present study, NfL remained elevated as compared to controls. Biphasic levels of serum GFAP were also observed, with lower levels in the first 6 months, then increasing over the remaining 5 years of the study (22), which differs from the present study. Although some similarities are observed with the present findings, the temporal profile of neuronal and glial blood biomarkers after TBIs, such as GFAP, NfL, and tau, remains to be fully understood, and further examination of serial biomarker assays in future work is warranted.

Blood-based biomarker measurement is a safe and accessible method to assess TBI, even years after injury, as well as related pathologic processes and potential neurobehavioral symptoms. Nonetheless, this study has several limitations. There was variability in certain group characteristics, as statistical power precluded differentiating into smaller groupings. In particular, the STBI group included participants with complicated mild, moderate, and severe TBI. Within the TBI groups, TBI mechanism of injury (i.e., blast vs. blunt force TBI, TBI sustained in deployment vs. prior to military service) may show different effects on biomarker concentrations. Study design also precluded the ability to account for potential confounders on blood biomarker levels, such as body mass index, glycosylated hemoglobin, renal function, and possible concomitant nervous system damage and systemic diseases (13). Further, a cross-sectional design does not allow for inference of a causal relationship between the biomarkers and neurobehavioral outcomes.

Despite these study limitations, findings suggest that decreased serum GFAP, NfL, and tau concentrations relate to worse neurobehavioral outcomes up to 5 years post-TBI, with a reverse trend observed at 8–10 years post-TBI. These findings were unexpected, and longitudinal GFAP and NfL concentrations should be further investigated through serial assays at chronic time points and methods with increased sensitivity, such as exosomes. Further studies are needed to help elucidate any patterns of association between blood-based biomarkers and neurobehavioral outcome over the course of the recovery trajectory following TBI.

## Data availability statement

The datasets presented in this article are not readily available because the data analyzed in this study is subject to the following licenses/restrictions: summary/aggregate data and additional information on the methods and statistical analyses will be provided on request. However, individual data elements are not available due to Department of Defense (DoD) legal requirements and current Institutional Review Board (IRB) approved language in the subject consent forms. Requests to access these datasets should be directed to RL, rael.lange@gmail.com.

## Ethics statement

The studies involving humans were approved by the Institutional Review Board of Walter Reed National Medical Military Center. The studies were conducted in accordance with the local legislation and institutional requirements. The participants provided their written informed consent to participate in this study.

## Author contributions

KE designed and conceptualized study, data collection, data analysis, and drafted the manuscript for intellectual content. SL, RL, LF, and TB designed and conceptualized the study, data collection, and revised the manuscript for intellectual content. KE and JG contributed to data analysis and revised the manuscript for intellectual content. All authors contributed to the article and approved the submitted version.

## Author disclaimer

The identification of specific products or scientific instrumentation is considered an integral part of the scientific endeavor and does not constitute endorsement or implied endorsement on the part of the author(s), DoD, or any component agency. The views expressed in this manuscript are those of the author(s) and do not necessarily reflect the official policy of the Department of Defense or the U.S. Government. This work was prepared under Contract HT0014-19-C-0004 with DHA Contracting Office (CO-NCR) HT0014 and, therefore, is defined as U.S. Government work under Title 17 U.S.C.§101. Per Title 17 U.S.C.§105, copyright protection is not available for any work of the U.S. Government. For more information, please contact dha.tbicoeinfo@health.mil.
